# A prognostic nomogram for patients with HR+ mucinous breast carcinoma based on the SEER database and a Chinese cohort study

**DOI:** 10.3389/fonc.2024.1444531

**Published:** 2024-08-23

**Authors:** Huiying Fang, Jian Yue, Hongzhong Li, Tiankuo Luan, Pin Wang, Guosheng Ren

**Affiliations:** ^1^ Chongqing Key Laboratory of Molecular Oncology and Epigenetics, The First Affiliated Hospital of Chongqing Medical University, Chongqing, China; ^2^ Department of Breast Cancer Center, Chongqing University Cancer Hospital, Chongqing, China; ^3^ Department of Breast Surgery, Gaozhou People’s Hospital, Gaozhou, China; ^4^ Center of Breast and Thyroid Surgery, Department of General Surgery, Chengdu Third People’s Hospital, Chengdu, China; ^5^ Department of Breast and Thyroid Surgery, The First Affiliated Hospital of Chongqing Medical University, Chongqing, China

**Keywords:** SEER, mucinous breast carcinoma, nomogram, propensity score matching analysis, prognosis, neoadjuvant chemotherapy

## Abstract

**Purpose:**

The study aimed to develop a nomogram model for individual prognosis prediction in patients with hormone receptors positive (HR+) mucinous breast carcinoma (MBC) and assess the value of neoadjuvant chemotherapy (NAC) in this context.

**Methods:**

A total of 6,850 HR+ MBC patients from the SEER database were identified and randomly (in a 7:3 ratio) divided into training cohorts and internal validation cohorts. 77 patients were enrolled from the Chongqing University Cancer Hospital as the external validation cohort. Independent risk factors affecting overall survival (OS) were selected using univariate and multivariate Cox regression analysis, and nomogram models were constructed and validated. A propensity score matching (PSM) approach was used in the exploration of the value of NAC versus adjuvant chemocherapy (AC) for long-term prognosis in HR+ MBC patients.

**Results:**

Multivariate Cox regression analysis showed 8 independent prognostic factors: age, race, marital status, tumor size, distant metastasis, surgery, radiotherapy, and chemotherapy. The constructed nomogram model based on these 8 factors exhibited good consistency and accuracy. In the training group, internal validation group and external validation group, the high-risk groups demonstrated worse OS (*p*<0.0001). Subgroup analysis revealed that NAC had no impact on OS (*p* = 0.18), or cancer specific survival (CSS) (*p* = 0.26) compared with AC after PSM.

**Conclusions:**

The established nomogram model provides an accurate prognostic prediction for HR+ MBC patients. NAC does not confer long-term survival benefits compared to AC. These findings provide a novel approach for prognostic prediction and clinical practice.

## 1 Introduction

Mucinous breast carcinoma (MBC) is a special subtype of breast cancer characterized by a significant presence of extracellular mucin. It has a low incidence rate, accounting for only 1% to 4% of all breast cancer cases (1, 2). The median age of onset for MBC is 68 years, which is older compared to invasive ductal carcinoma (IDC) (3). Importantly, MBC generally carries a more favorable prognosis (4, 5). The majority of MBC belong to the Luminal type (6), with over 90% positive expression of hormone receptors (HR) (7, 8). The majority of MBC cases exhibit HR positivity, which generally confers a better prognosis compared to HR- MBC. According to the NCCN guidelines, the treatment of HR-positive (HR+) MBC is different from that of HR-negative (HR-) patients. Despite many new advancements in tumor treatment (9, 10), treatment strategies for HR+ MBC primarily involve chemotherapy and hormonal therapy (11), whereas HR- MBC treatment aligns more closely with triple-negative breast cancer, focusing predominantly on chemotherapy. Previous research on MBC has predominantly centered on HR+ MBC cohorts, with minimal investigation into HR- MBC. Furthermore, numerous studies have demonstrated that HR status is an independent prognostic factor influencing the outcomes of MBC (4, 12). To mitigate bias in our data analysis and better study MBC, we excluded HR- MBC data, opting solely for HR+ MBC, which better represents the biological and prognostic characteristics of MBC. Therefore, the focus of this study is to specifically investigate the HR+ MBC population.

Due to the relatively small number of patients and significant biological differences compared to IDC, there are no specific diagnostic and treatment guidelines for MBC currently. In the existing guidelines and clinical practice, surgery and adjuvant therapy are still the main treatment strategies for HR+ MBC. Because mucinous carcinoma grows slowly and is often diagnosed with a large mass (13), neoadjuvant chemotherapy (NAC) is often involved in clinical treatment. However, whether NAC has long-term survival benefits for MBC than adjuvant chemotherapy (AC) has not yet been confirmed by studies. Therefore, the value of NAC of MBC is a clinical question that needs to be evaluated.

Currently, the development of bioinformatics has significantly contributed to advancing prognostic prediction and tumor treatment (14). However, there is a lack of reliable prognostic evaluation systems for personalized treatment of HR+ MBC. Previous study have demonstrated certain method for predicting breast cancer survival (15). Nomogram as a novel cancer prediction model, has the ability to identify and stratify clinical patients on an individual basis (16–18). It offers a more accurate and intuitive approach compared to traditional TNM staging and has emerged as a new standard for tumor prognosis prediction. The objective of our study is to develop a nomogram model based on clinicopathological features to predict the prognosis of HR+ MBC and to evaluate the benefit of NAC in HR+ MBC.

## 2 Methods

### 2.1 Study design and data sources

**Figure 1:**
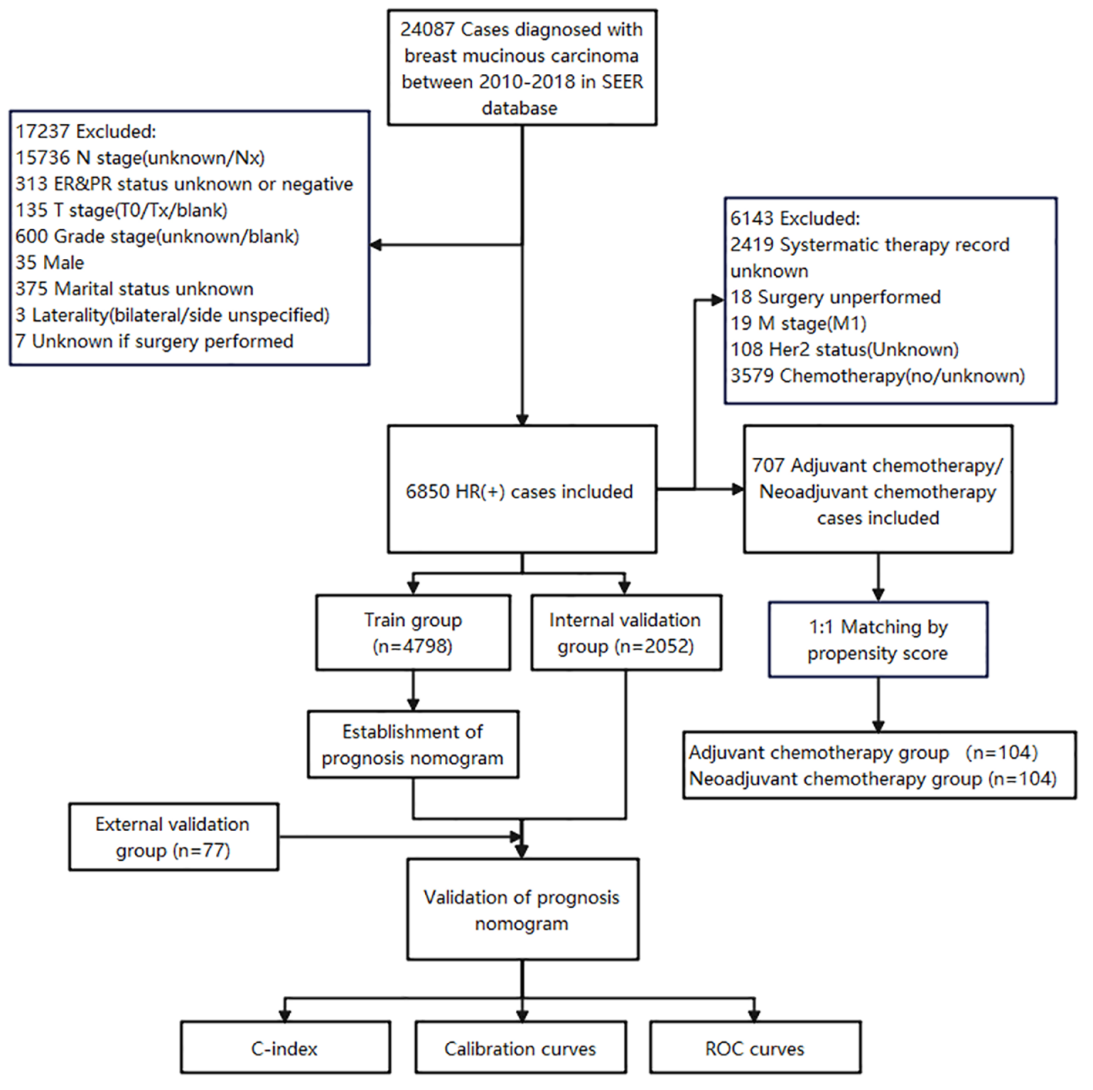
Flowchart for patients' selection.

The data for this study were obtained from the Surveillance, Epidemiology, and End Results (SEER) database of the US National Cancer Institute (NCI), and the data were obtained by SEER Stat 8.4.1 software. 24087 cases diagnosed with MBC between 2010-2018 in SEER database were initially collected. The inclusion criteria were as follows (1): ICD-O-3, Hist/behave, malignant = “8480/3: mucinous adenocarcinoma”; (2) hormone receptor positive (HR+) (includes estrogen receptor and progesterone receptor); (3) MBC as the first and only cancer diagnosis; (4) patients with complete general clinicopathological information; (5) well-established follow-up to ensure reliable patient status. The exclusion criteria were given as follows: (1) male patients; (2) patients with bilateral breast cancer; (3) patients with missing information (N stage, T stage, Grade stage, HR/Her2 status, marital status and surgical experience) (**Figure 1**).

### 2.2 Cohort definition and information extraction

6850 HR+ MBC patients were randomly assigned to the training cohort and validation cohort according to a ratio of 7:3. The external validation cohort was comprised of HR+ MBC patients treated at the Chongqing University Cancer Hospital between 2012 and 2019. The diagnosis of hormone receptor status is determined by evaluating the immunohistochemistry results of the patient’s lesion. We applied identical eligibility criteria as those utilized for the patients extracted from the SEER database, culminating in the enrollment of 77 patients. Follow-up procedures were conducted through telephone interviews, follow-up of more than 5 years with the final follow-up date recorded as May 10, 2024. All patients have obtained follow-up results. This study is a retrospective analysis grounded in clinical data, informed consent from patients was not necessitated.

The training cohort is used to build models and filter variables, and the validation cohort is used to verify the models. The following variables was extracted from SEER: age, race, marital status, laterality, histological grade, tumor size, axillary lymph nodes, metastatic status, human epidermal growth factor receptor 2 (HER2) status, surgery, radiotherapy, chemotherapy, survival data. Similarly, external validation data were collected for the clinical variables mentioned above and corresponding statistical analyses were conducted. It is worth noting that, due to the Chinese origin of the external data, the race category only includes the “other” class.

### 2.3 Statistical analysis

Statistical analysis was performed using R version 4.3.0. Differences in clinical characteristics of patients in the training and validation cohorts were compared using the chi-square test. Using univariate and multivariate Cox analysis, independent prognostic factors (*p* < 0.05) were identified, and then the nomogram prognostic models of MBC 3-year and 5-year overall survival (OS) rates were constructed using the selected variables. Concordance index (C-index) and area under the receiver operating characteristic (ROC) curve were used to evaluate discriminative ability. C-index and ROC values range between 0.5 - 1, where 0.5 means no predictive ability, 1.0 means full accuracy. An estimate that is greater than 0.7 is usually considered reasonable. The mode’s accuracy is evaluated by the calibration curve. Model accuracy increases as the actual probability line approaches the reference line.

In order to reduce data selection bias and confounding factors, we used a propensity score-matched (PSM) analysis of patients receiving NAC and AC, respectively (caliper = 0.05). 10 variables including age, race, marital status, laterality, grade, tumor size, axillary lymph nodes, surgery, radiotherapy, and luminal subtype that may affect HR+ MBC patients’ survival were selected for matching, and a ratio of 1:1 was obtained. OS and cancer-specific survival (CSS) was determined using the Kaplan-Meier and log-rank tests. Two-sided *p* values < 0.05 were considered statistically significant.

## 3 Results

### 3.1 Patients’ baseline characteristics

This study included a total of 6850 HR+ MBC cases from SEER database, with 4798 cases (70.0%) in the training cohort and 2052 cases (30.0%) in the internal validation cohort. The two groups of patients showed no differences in clinical pathological and demographic characteristics. The majority of patients were ≥ 50 years old (87.6%) and white (75.8%), and there were no significant differences in marital status or breast cancer affected side. Most cases had favorable histological grading, with grade I accounting for 59.2% and grade II accounting for 37.2%. Breast masses at diagnosis were generally ≤ 5 cm, with T1 accounting for 66.8%, T2 for 27.2%, T3 for 4.6%, and T4 for only 1.4%. Most patients did not have axillary lymph node metastases (91.6%), indicating a mild biological behavior of HR+ MBC. The majority of patients were HER2-negative (92.4%), while only 322 patients (4.7%) were HER2-positive. 6600 patients (92.4%) received surgery, and 793 patients (11.6%) received chemotherapy, including NAC and AC. The external validation cohort from the Chongqing University Cancer Hospital had 77 patients. All patients were Asian females, with 98.7% being married. The distribution of patients aged ≥50 years and <50 years was roughly equivalent. The vast majority presented with HER2-negative breast cancer and without distant metastasis. Surgical treatment was administered to 92.2% of the patients, while 26.0% underwent radiotherapy and 83.1% received chemotherapy. The clinicopathological characteristics of all patients are outlined in **Table 1**.

**Table 1:** Demographics and pathological characteristics of patients with mucinous breast carcinoma.

Variables	Overall (n=6850) n (%)	Training cohort (n=4798) n (%)	Internal validation cohort (n=2052) n (%)	External validation cohort (n=77) n (%)	T vs IV (p value)	T vs EV (p value)
**Age**					0.470	<0.001
<50	845 (12.4)	601 (12.6)	244 (11.9)	43 (55.8)		
≥50	5995 (87.6)	4187 (87.4)	1808 (88.1)	34 (44.2)		
**Race**					0.284	<0.001
White	5185 (75.8)	3606 (75.3)	1579 (76.9)	0 (0.0)		
Black	836 (12.2)	591 (12.3)	245 (11.9)	0 (0.0)		
Other	819 (12.0)	591 (12.3)	228 (11.1)	77 (100.0)		
**Marital**					0.712	<0.001
Married	3445 (50.4)	2404 (50.2)	1041 (50.7)	76 (98.7)		
Single	3395 (49.6)	2384 (49.8)	1011 (49.3)	1 (1.3)		
**Laterality**					0.870	0.206
Left	3478 (50.8)	2431 (50.8)	1047 (51.0)	33 (42.9)		
Right	3362 (49.2)	2357 (49.2)	1005 (49.0)	44 (57.1)		
**Grade**					0.441	<0.001
I	4048 (59.2)	2813 (58.8)	1235 (60.2)	29 (37.7)		
II	2544 (37.2)	1804 (37.7)	740 (36.1)	40 (51.9)		
III	248 (3.6)	171 (3.6)	77 (3.8)	8 (10.4)		
**T**					0.939	<0.001
1	4571 (66.8)	3196 (66.8)	1375 (67.0)	18 (23.4)		
2	1861 (27.2)	1309 (27.3)	552 (26.9)	42 (54.5)		
3	314 (4.6)	216 (4.5)	98 (4.8)	13 (16.9)		
4	94 (1.4)	67 (1.4)	27 (1.3)	4 (5.2)		
**N**					0.163	<0.001
0	6267 (91.6)	4407 (92.0)	1860 (90.6)	58 (75.3)		
1	470 (6.9)	317 (6.6)	153 (7.5)	8 (10.4)		
2	68 (1.0)	41 (0.9)	27 (1.3)	5 (6.5)		
3	35 (0.5)	23 (0.5)	12 (0.6)	6 (7.8)		
**M**					1.000	0.004
0	6768 (98.9)	4738 (99.0)	2030 (98.9)	73 (94.8)		
1	72 (1.1)	50 (1.0)	22 (1.1)	4 (5.2)		
**HER2**					0.687	0.821
Negative	6316 (92.3)	4428 (92.5)	1888 (92.0)	71 (92.2)		
Positive	322 (4.7)	224 (4.7)	98 (4.8)	3 (3.9)		
Unknown	202 (3.0)	136 (2.8)	66 (3.2)	3 (3.9)		
**Surgery**					0.757	0.116
No	249 (3.6)	177 (3.7)	72 (3.5)	6 (7.8)		
Yes	6591 (96.4)	4611 (96.3)	1980 (96.5)	71 (92.2)		
**Radiation**					0.353	<0.001
No	3553 (51.9)	2469 (51.6)	1084 (52.8)	57 (74.0)		
Yes	3287 (48.1)	2319 (48.4)	968 (47.2)	20 (26.0)		
**Chemotherapy**					0.705	<0.001
No	6047 (88.4)	4238 (88.5)	1809 (88.2)	13 (16.9)		
Yes	793 (11.6)	550 (11.5)	243 (11.8)	64 (83.1)		

HER2, human epidermal growth factor receptor 2.

### 3.2 Identification of risk factors for HR+ MBC

We conducted both univariate and multifactorial Cox regression analyses to identify the risk factors that affect OS. The results of the univariate analysis showed that age, race, marital status, T stage, metastasis, surgery, radiotherapy, and chemotherapy were all significantly associated with OS (*p* < 0.05). Additionally, the multivariate analysis identified age, race, marital status, mass size, metastasis, and treatment as independent prognostic factors for HR+ MBC in the nomogram model. The Cox regression survival analysis based on OS is presented in **Table 2**.

**Table 2:** Univariate and Multivariate Analysis of Overall Survival in the Training Group.

Variables	Univariate analysis		Multivariate analysis	
	HR (95% CI)	p value	HR (95% CI)	p value
Age				
<50	Reference		Reference	
≥50	6.39 (3.89 - 10.5)	0	4.99 (3.01 - 8.25)	0
Race				
White	Reference		Reference	
Black	0.88 (0.69 - 1.11)	0.280	0.79 (0.62 - 1)	0.0492
Other	0.45 (0.33 - 0.62)	0	0.56 (0.41 - 0.77)	0.0004
Marital				
Married	Reference		Reference	
Single	2.10 (1.79 - 2.46)	0	1.73 (1.47 - 2.04)	0
Laterality				
Left	Reference		–	–
Right	1.01 (0.86 - 1.17)	0.948	–	–
Grade				
I	Reference		–	–
II	0.99 (0.84 - 1.16)	0.869	–	–
III	1.44 (1 - 2.07)	0.051	–	–
T				
1	Reference		Reference	
2	1.64 (1.39 - 1.94)	0	1.59 (1.35 - 1.89)	0
3	1.93 (1.42 - 2.63)	0	1.65 (1.18 - 2.3)	0.0031
4	4.75 (3.19 - 7.07)	0	2.46 (1.51 - 4.01)	0.0003
N				
0	Reference		Reference	
1	1.18 (0.89 - 1.56)	0.263	1.20 (0.88 - 1.63)	0.242
2	1.21 (0.58 - 2.56)	0.611	1.42 (0.78 - 3.81)	0.1793
3	2.40 (1.19 - 4.82)	0.014	0.67 (0.3 - 1.52)	0.3399
M				
0	Reference		Reference	
1	8.55 (5.98 - 12.21)	0	2.23 (1.38 - 3.61)	0.001
Surgery				
No	Reference		Reference	
Yes	0.15 (0.12 - 0.19)	0	0.30 (0.23 - 0.39)	0
Radiation				
No	Reference		Reference	
Yes	0.43 (0.37 - 0.51)	0	0.55 (0.46 - 0.65)	0
Chemotherapy				
No	Reference		Reference	
Yes	0.42 (0.30 - 0.57)	0	0.46 (0.32 - 0.66)	0
HER2				
Negative	Reference		Reference	
Positive	0.65 (0.43 - 1.00)	0.048	0.92 (0.6 - 1.43)	0.7213
Unknown	1.22 (0.84 - 1.78)	0.290	1.12 (0.77 - 1.64)	0.5445

HR, hazard ratio; CI, confidence interval; HER2, human epidermal growth factor receptor 2.

### 3.3 Nomogram construction and validation

**Figure 2:**
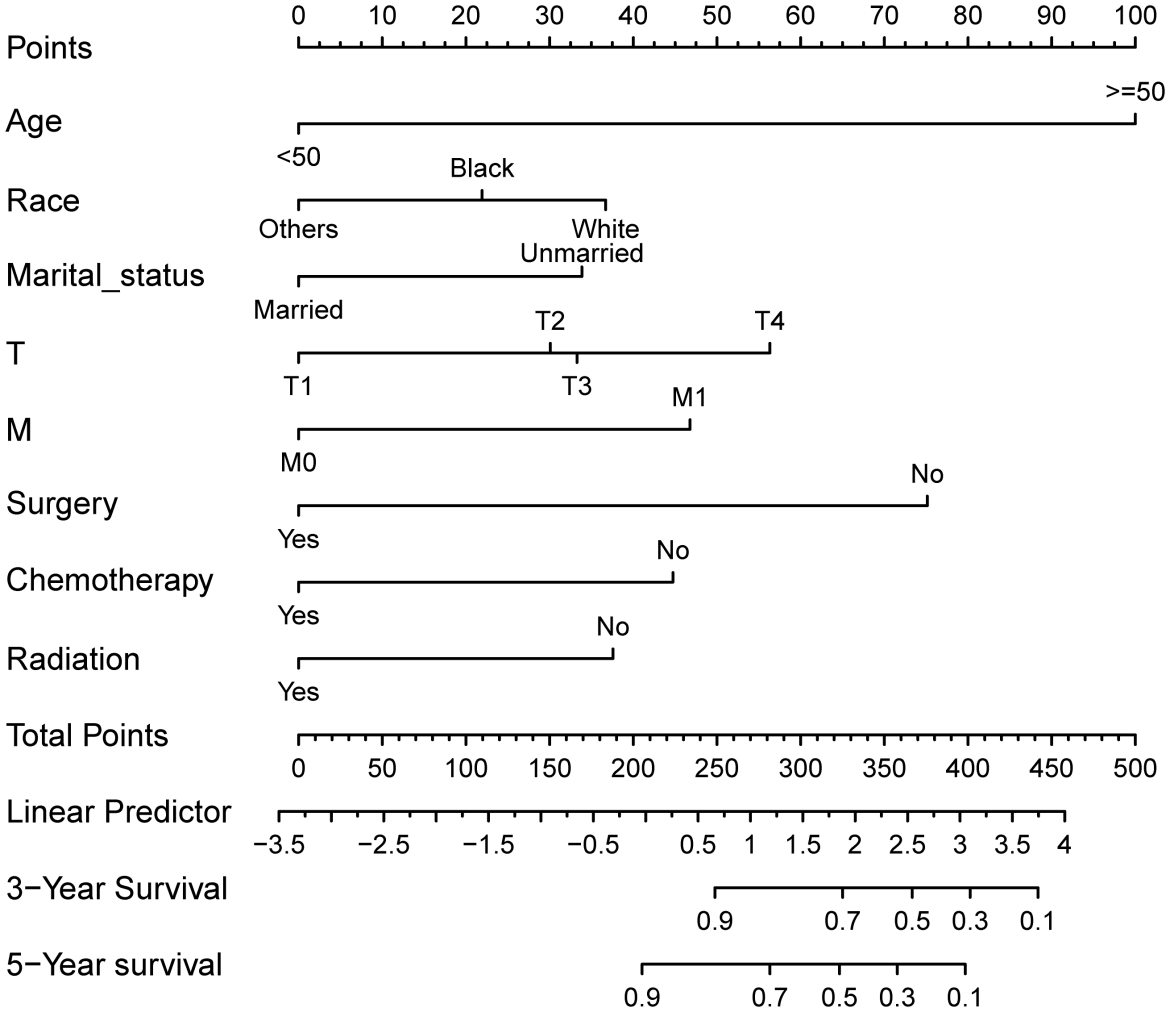
Nomogram model for prognostic prediction of HR^+^ MBC patients. Each variable is quantified as a different value corresponding to a point at the top of the chart, the sum of all variable values represents the total number of points on the bottom scale, reflecting the overall survival rate at 3- and 5- years, respectively.

A visual nomogram model was developed based on the prognostic risk factors identified from Cox analysis. Each variable was assigned a score ranging from 1 to 100 based on its corresponding coefficient. The scores for each variable were then summed to obtain a total score, which was associated with the 3-year and 5-year OS rates. Higher scores indicate lower OS rates. The nomogram prediction plots are presented in **Figure 2**.

**Figure 3:**
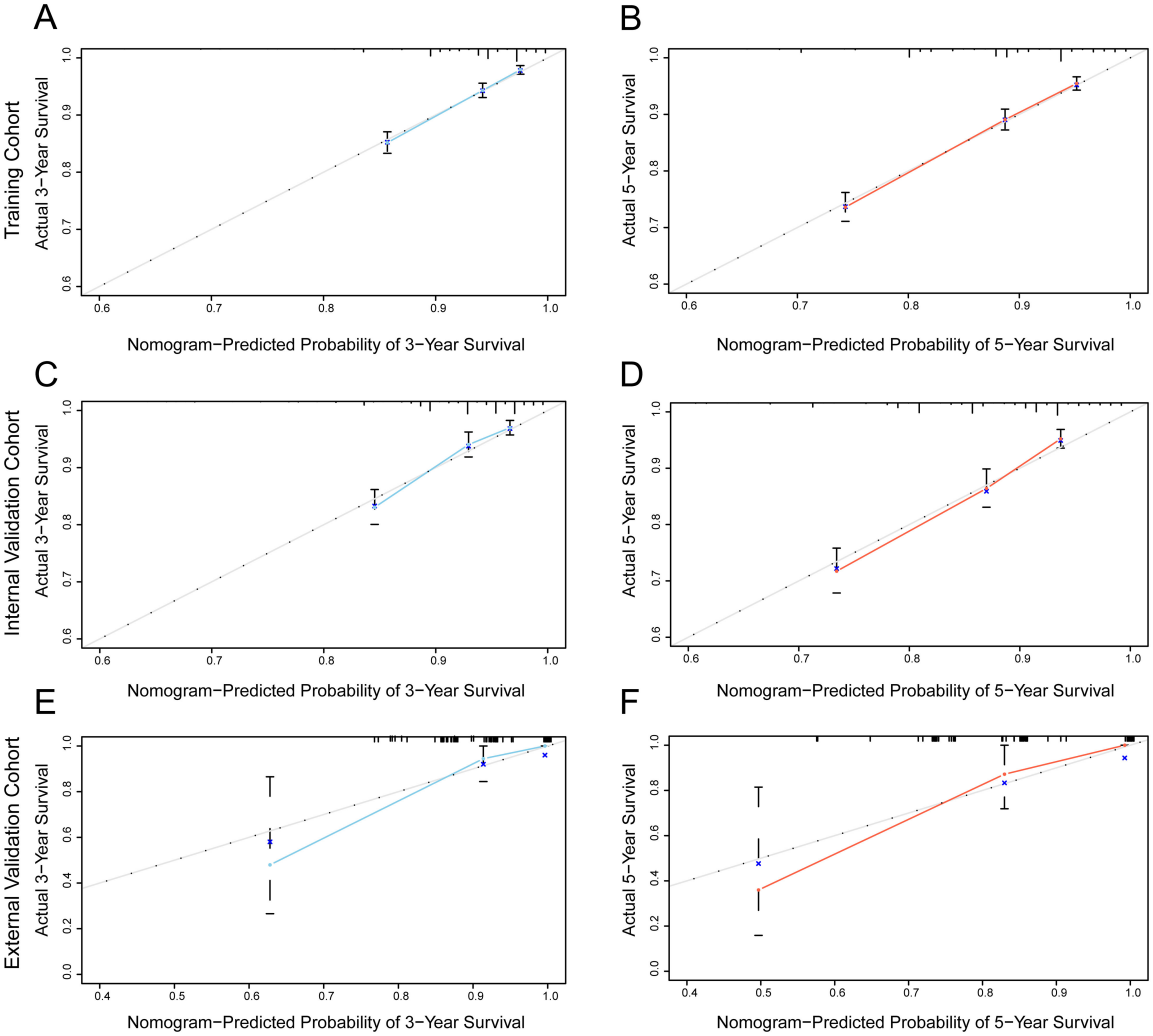
Calibration curve of the nomogram for predicting the probability of 3-year and 5-year OS of MBC. **(A, B)** training cohort; **(C, D)** internal validation cohort; **(E, F)** external validation cohort.

The predictive ability of the nomogram model was evaluated by calculating the C-index using R software. For the training cohort, the C-index was 0.735 (95% CI: 0.7154 ~ 0.7546), for the internal validation cohort, it was 0.733 (95% CI: 0.7036 ~ 0.7624), and for the external validation cohort, it was 0.967 (95% CI: 0.938 ~ 0.996). These results suggest that the model accurately assesses prognosis. The calibration plots revealed that the calibration curves for the training cohort as well as the internal and external validation cohorts, pertaining to both 3-year and 5-year OS predictions, closely adhered to the ideal 45° reference line as depicted in **Figure 3**. It demonstrated that the predicted values are in good consistency with the actual 3-year and 5-year OS rates.

**Figure 4:**
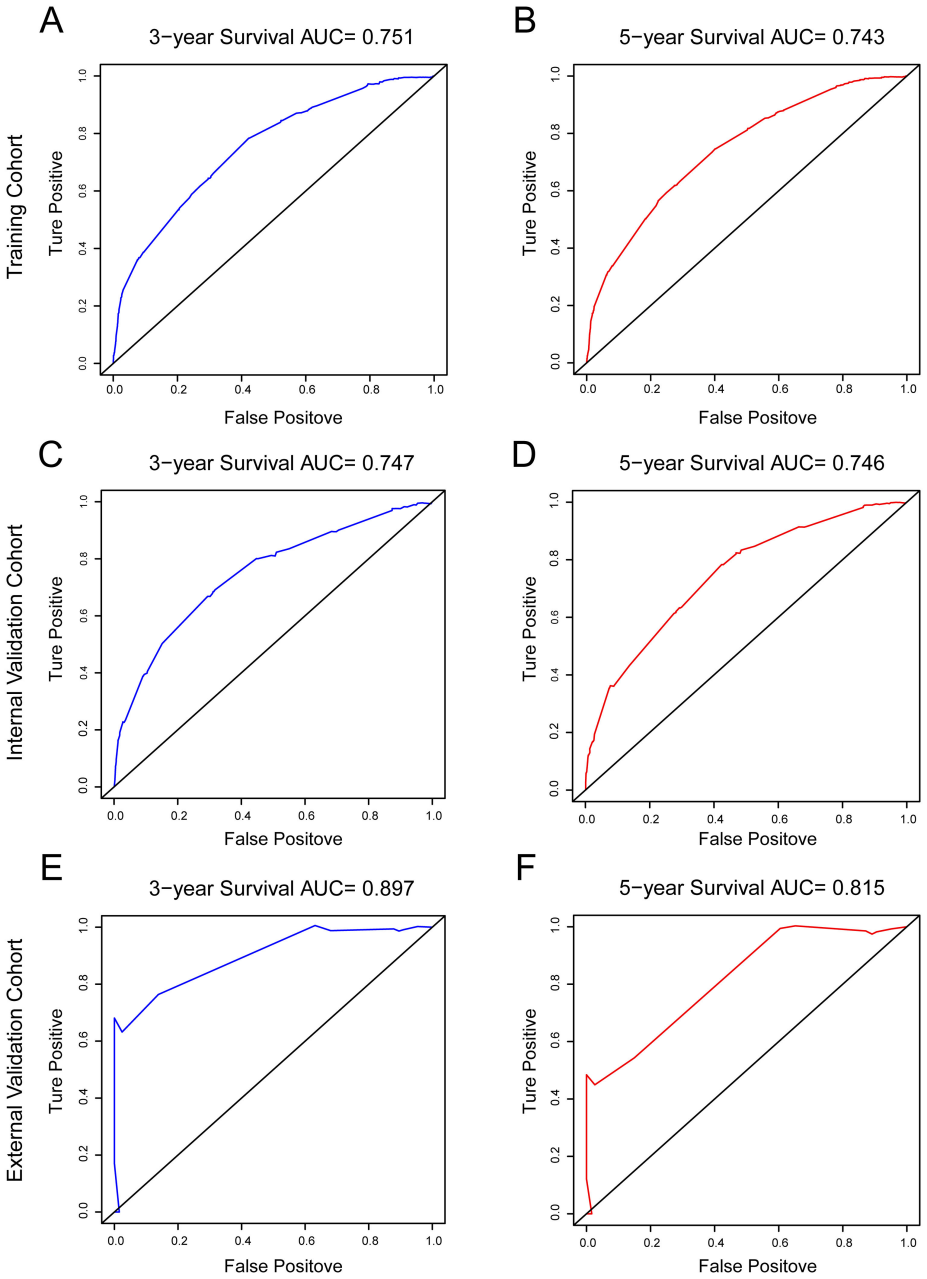
ROC curves were used to test the prediction ability of the model for 3-, and 5-year overall survival. **(A, B)** training cohort; **(C, D)** internal validation cohort; **(E, F)** external validation cohort.

The ROC curves were performed to predict 3-year and 5-year OS, and areas under the curve (AUCs) were all > 0.7 in the training, internal and external validation cohorts in **Figure 4**. It indicating that the nomogram exhibits excellent discriminatory ability. In conclusion, the model improved the estimation of OS in HR+ MBC and provide guidance for clinical prognosis evaluation and therapeutic decision-making.

### 3.4 Survival analysis for OS

**Figure 5:**
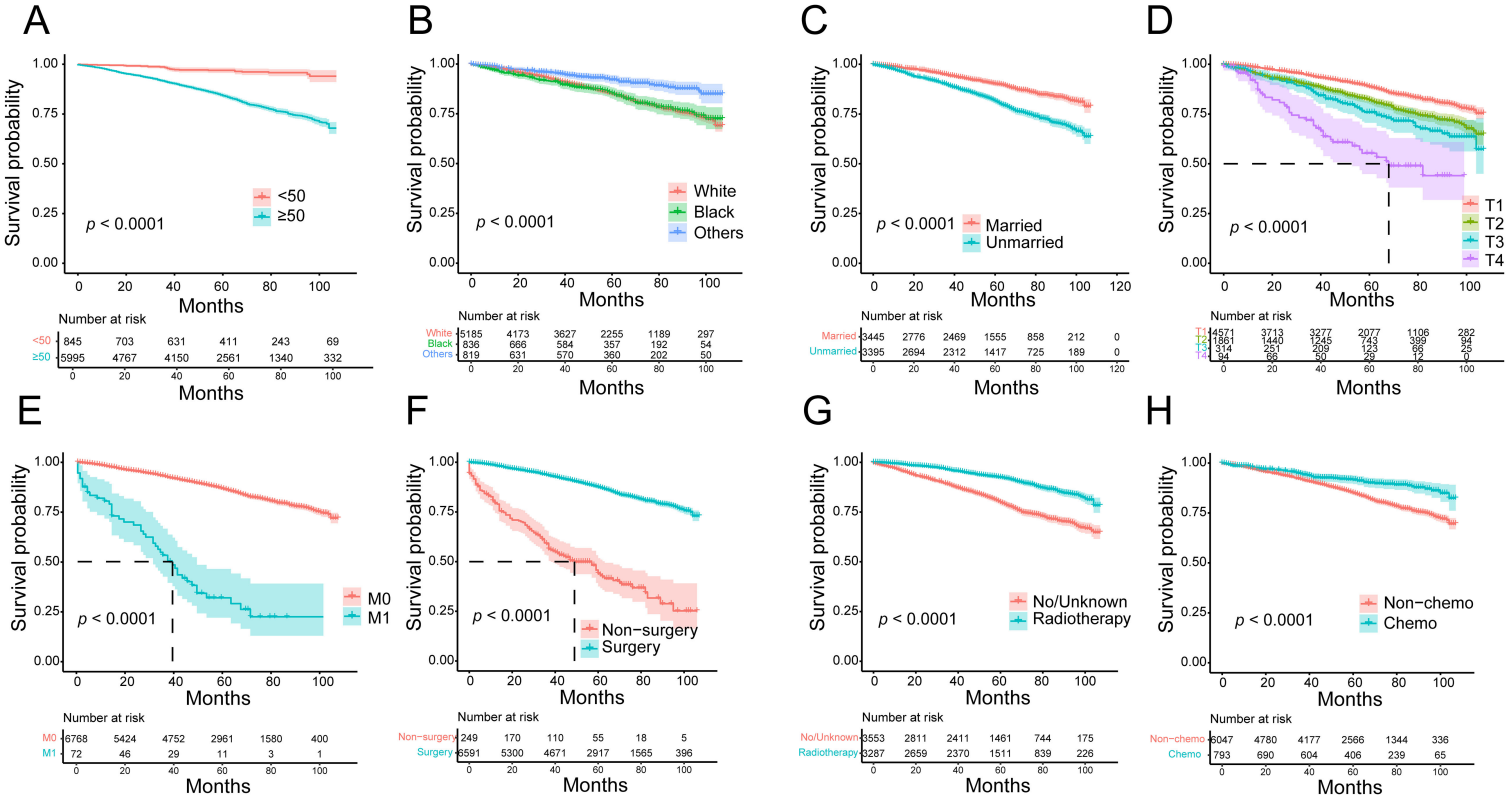
Kaplan-Meier survival curves for overall survival in HR^+^ MBC patients. **(A)** Age; **(B)** race; **(C)** marital status; **(D)** T stage; **(E)** metastasis status; **(F)** surgery; **(G)** radiotherapy; **(H)** chemotherapy.

To further confirm the impact of risk predictor stratification on OS in HR+ MBC, we performed a Kaplan-Meier survival analysis. Patients aged ≥ 50 years (*p* < 0.0001, HR = 5.559, 95% CI [4.631-6.673]), unmarried (*p* < 0.0001, HR = 1.962, 95% CI [1.731-2.223]), M1 (*p* < 0.0001, HR = 8.128, 95% CI [3.651-18.095]), and those with an advanced T-stage (*p* < 0.0001, HR = 1.163 [1.413-1.887]) had lower OS rates; surgery (*p* < 0.0001, HR = 0.158, 95% CI [0.105-0.236]), chemotherapy (*p* < 0.0001, HR = 0.529, 95% CI [0.440-0.637]), and radiotherapy (*p* < 0.0001, HR = 0.413, 95% CI [0.364-0.468]) increased OS benefit (**Figure 5**).

**Figure 6:**
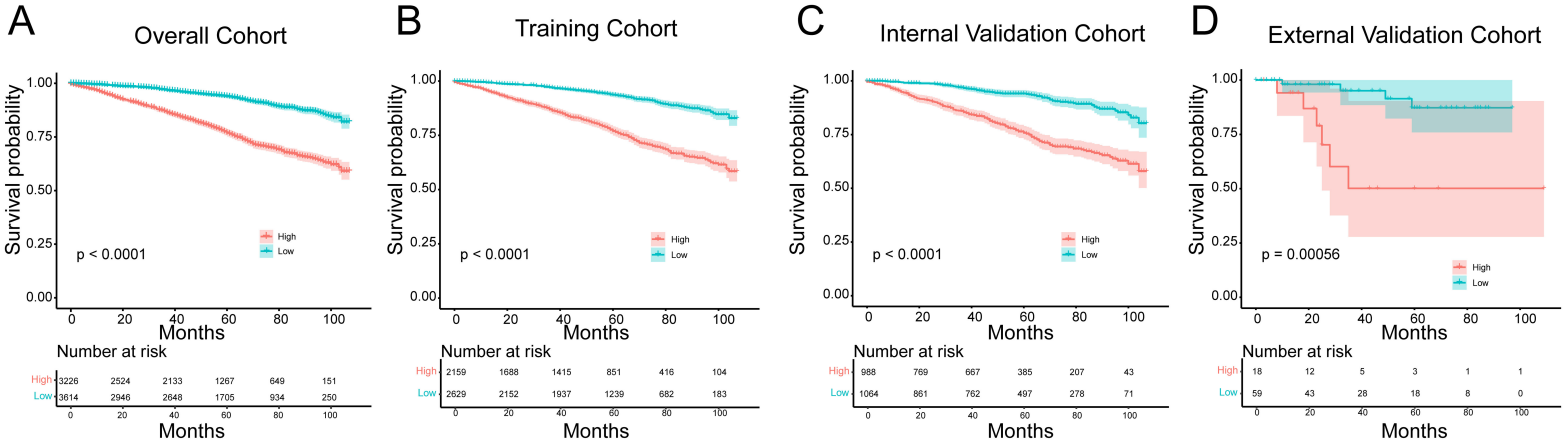
Kaplan-Meier survival curves for the low and high- risk groups in the **(A)** overall cohort, **(B)** training cohort, **(C)** internal validation cohort and **(D)** external validation cohort.

Finally, based on the nomogram model, we calculated the linear predicted values corresponding to the total scores of each patient of training cohort, using the median risk score as the cut-off value to allocate patients into high-risk group and a low-risk group. Kaplan-Meier curves demonstrated significantly better OS in the low-risk group compared to the high-risk group in the overall cohort (*p* < 0.0001, HR = 0.287, 95% CI [0.253-0.326]), training cohort (*p* < 0.0001, HR = 0.280, 95% CI [0.241-0.327]), internal validation cohort (*p* < 0.0001, HR = 0.284, 95% CI [0.227-0.356]) and external validation cohort (*p* = 0.00056, HR = 0.148, 95% CI [0.030-0.739]) (**Figure 6**). This suggests that the nomogram model can provide accurate risk stratification for HR+ MBC patients.

### 3.5 Prognostic value of neoadjuvant chemotherapy in HR+ MBC

**Figure 7:**
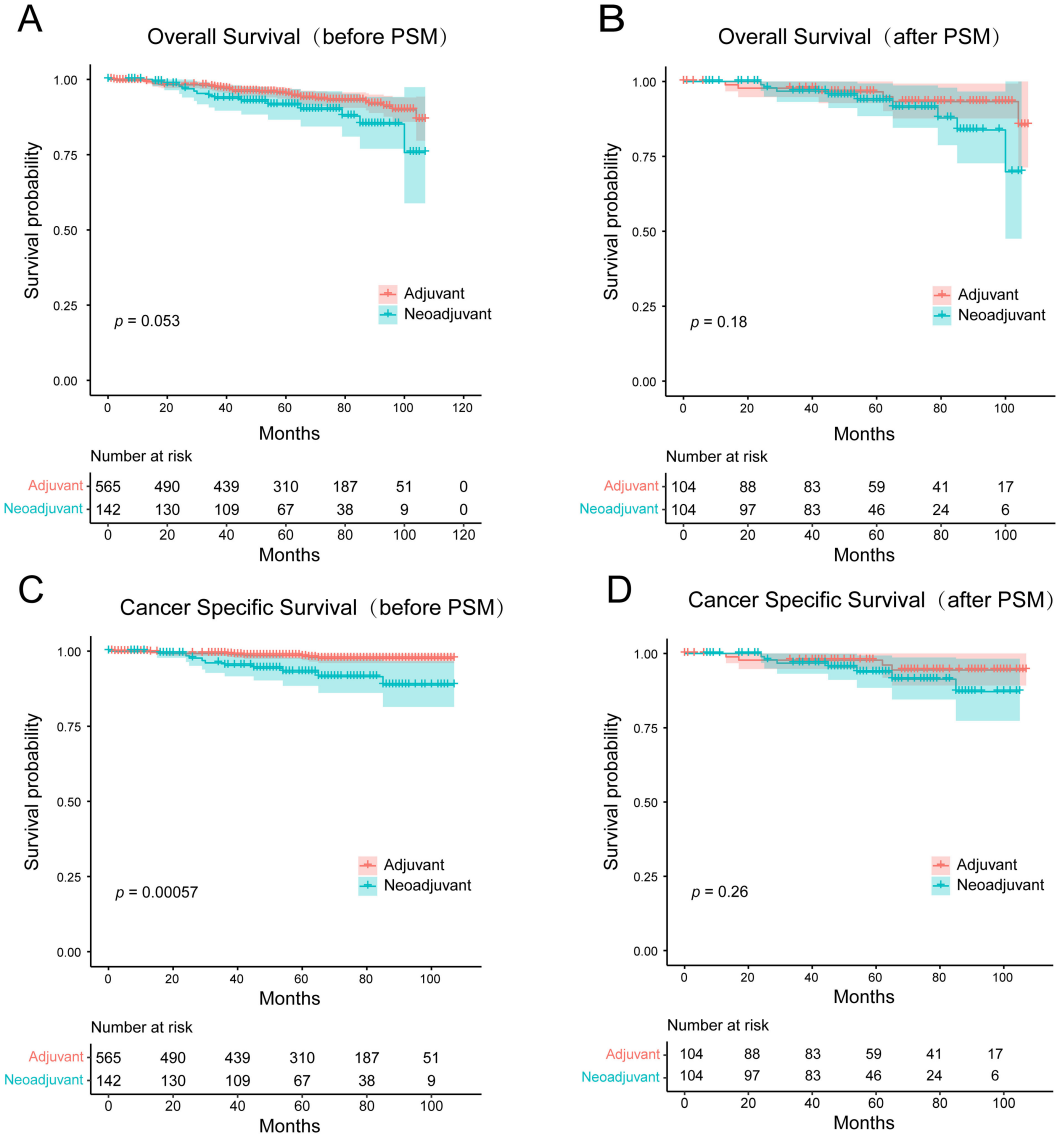
Kaplan-Meier survival curves for **(A, B)** overall survival and **(C, D)** cancer-specific survival of the adjuvant chemotherapy and the neoadjuvant chemotherapy groups before and after PSM.

NAC has been shown to improve survival in some breast cancers such as triple negative and HER2-positive breast cancers, but its value in MBC has not been confirmed in studies. To mitigate selection bias and confounding factors, we conducted PSM analysis on a cohort of 707 HR+ MBC patients who received NAC or AC. The details of the original and matched cohorts before and after PSM are presented in **Table 3**. Before PSM, there was no statistical difference in OS between patients who underwent NAC and AC (*p* = 0.053, HR = 1.833, 95% CI [0.882-3.811]), and patients receiving NAC had worse CSS (*p* = 0.00057, HR = 4.128, 95% CI [1.366-12.478]). But after 1:1 matching, there was no difference in OS (*p* = 0.18, HR = 1.973, 95%CI [0.710-5.480]) and CSS (*p* = 0.26, HR = 1.994, 95% CI [0.610-6.517]) between the two groups (**Figure 7**). These findings suggest that NAC does not confer a survival benefit in HR+ MBC.

**Table 3:** The baseline characteristics of patients undergoing chemotherapy before and after PSM.

Variables	Before PSM			After PSM		
	Adjuvant chemotherapy (n=565)	Neoadjuvant chemotherapy (n=142)	P value	Adjuvant chemotherapy (n=104)	Neoadjuvant chemotherapy (n=104)	P value
**Age**			0.0221			0.573
<50	190 (33.6%)	63 (44.4%)		40 (38.5%)	45 (43.3%)	
≥50	375 (66.4%)	79 (55.6%)		64 (61.5%)	59 (56.7%)	
**Race**			0.966			0.701
White	383 (67.8%)	96 (67.6%)		73 (70.2%)	71 (68.3%)	
Black	95 (16.8%)	25 (17.6%)		19 (18.3%)	17 (16.3%)	
Other	87 (15.4%)	21 (14.8%)		12 (11.5%)	16 (15.4%)	
**Marital**			0.019			0.675
Married	350 (61.9%)	72 (50.7%)		61 (58.7%)	57 (54.8%)	
Single	215 (38.1%)	70 (49.3%)		43 (41.3%)	47 (45.2%)	
**Laterality**			0.311			0.674
Left	284 (50.3%)	64 (45.1%)		42 (40.4%)	46 (44.2%)	
Right	281 (49.7%)	78 (54.9%)		62 (59.6%)	58 (55.8%)	
**Grade**			0.713			0.75
I	204 (36.1%)	52 (36.6%)		34 (32.7%)	39 (37.5%)	
II	287 (50.8%)	75 (52.8%)		56 (53.8%)	51 (49.0%)	
III	74 (13.1%)	15 (10.6%)		14 (13.5%)	14 (13.5%)	
**T**			<0.001			0.914
1	286 (50.6%)	23 (16.2%)		24 (23.1%)	22 (21.2%)	
2	223 (39.5%)	48 (33.8%)		42 (40.4%)	47 (45.2%)	
3	48 (8.5%)	53 (37.3%)		32 (30.8%)	30 (28.8%)	
4	8 (1.4%)	18 (12.7%)		6 (5.8%)	5 (4.8%)	
**N**			<0.001			0.95
0	394 (69.7%)	55 (38.7%)		51 (49.0%)	50 (48.1%)	
1	139 (24.6%)	61 (43.0%)		37 (35.6%)	39 (37.5%)	
2	21 (3.7%)	18 (12.7%)		11 (10.6%)	9 (8.7%)	
3	11 (1.9%)	8 (5.6%)		5 (4.8%)	6 (5.8%)	
**Stage**			<0.001			0.96
I	272 (48.1%)	16 (11.3%)		17 (16.3%)	16 (15.4%)	
II	241 (42.7%)	62 (43.7%)		57 (54.8%)	59 (56.7%)	
III	52 (9.2%)	64 (45.1%)		30 (28.8%)	29 (27.9%)	
**Radiation**			0.0215			0.316
No	262 (46.4%)	50 (35.2%)		35 (33.7%)	43 (41.3%)	
Yes	303 (53.6%)	92 (64.8%)		69 (66.3%)	61 (58.7%)	
**Subtype**			0.362			0.74
Luminal A	433 (76.6%)	103 (72.5%)		82 (78.8%)	79 (76.0%)	
Luminal B	132 (23.4%)	39 (27.5%)		22 (21.2%)	25 (24.0%)	

PSM, propensity score matching.

## 4 Discussion

Mucinous breast carcinoma is a distinct and rare subtype of breast cancer characterized by unique clinicopathological features. Compared to invasive ductal carcinoma, MBC exhibits a more favorable prognosis (19, 20). In our study, we conducted Cox analysis to identify eight risk factors that influence the prognosis of HR+ MBC. These factors include age, race, marital status, tumor size, distant metastasis, surgery, radiotherapy, and chemotherapy. Our results revealed that a majority of HR+ MBC patients (87.6%) were initially diagnosed at an advanced age (≥50 years). Importantly, we observed that patients below 50 years of age exhibited better OS outcomes compared to those aged 50 years and above, consistent with previous research findings (5, 12, 21). Furthermore, our study found that white and black individuals had a higher risk of mortality than other races, which aligns with the findings reported by Zhu et al. (21). This suggests the existence of racial disparities in the prognosis of HR+ MBC, with Asian populations experiencing a lower prognostic risk (12).

The influence of tumor size on the prognosis of MBC has been a topic of debate. While the National Comprehensive Cancer Network (NCCN) guidelines recommend the consideration of axillary lymph node metastasis rather than mass size when determining adjuvant chemotherapy for HR+ MBC, the effect of tumor size remains uncertain. J.C. Paramo et al. concluded that mass size was not associated with lymph node metastasis, raising concerns about its significance in predicting prognosis (22). However, some investigators suggested that the masses are larger in lymph node positive patients (23). This ambiguity arises due to the unique structural characteristics of mucinous carcinoma masses, which contain large pools of mucus (4, 24). As a result, the actual size of the mass does not accurately represent the tumor boundaries, making it challenging to delineate the precise size of the tumor lesion (8, 25). However, our multivariate analysis of HR+ MBC demonstrated that tumor size independently affects prognosis. Specifically, larger masses were associated with lower OS rates as the previous study has shown (12), indicating the potential for improved prognosis through early detection and resection. These findings emphasize the importance of timely intervention in HR+ MBC cases. Interestingly, married patients exhibit superior OS compared to unmarried patients, which may be attributed to a later age at diagnosis and the receipt of more comprehensive treatment and care among married individuals. Furthermore, MBC patients underwent surgery, radiotherapy and chemotherapy exhibited prolonged OS, particularly those who underwent surgery. This highlights surgery as the paramount therapeutic approach for MBC patients, while concurrent adjunct therapies such as chemotherapy and radiotherapy offer maximal survival benefits.

Currently, a systematic and personalized treatment guideline for mucinous carcinoma is lacking. The main treatment modalities for this subtype include endocrine therapy and chemotherapy. In cases which axillary lymph nodes are negative or micro-metastasis (≤ 2mm), endocrine therapy is recommended. For patients with positive axillary lymph nodes, a combination of adjuvant endocrine therapy and chemotherapy is recommended (26, 27). However, the indications for NAC and radiotherapy remain unclear in HR+ MBC. In the context of invasive ductal carcinoma, radiotherapy plays a crucial role in breast-conserving surgery and high-risk patients (28, 29). Previous studies have demonstrated that both postoperative adjuvant chemotherapy and radiotherapy improve the prognosis of MBC (27, 30–32). In our study, a considerable proportion of patients (48.0%) received radiotherapy, while 11.6% underwent chemotherapy, indicating significant improvements in OS with the use of both treatments. Notably, while some previous studies have identified lymph node metastasis as a prognostic risk factor (33–35), our findings did not reveal any significant differences, consistent with the results of Zhu et al. (21). This discrepancy can be attributed to the enrollment of predominantly estrogen receptor-positive patients with a favorable prognosis, along with a high proportion (91.6%) of lymph node-negative individuals, which diminished the risk stratification associated with lymph node metastasis.

NAC is commonly administered to patients with IDC who present with large tumor masses, as well as those with triple-negative and HER2-positive subtypes. Extensive clinical studies have demonstrated its prognostic benefits in these cases (36–40). However, the value and indications of NAC in HR+ MBC have been rarely reported. To explore the potential benefits of NAC in HR+ MBC, we conducted a subgroup analysis focusing on patients who received either NAC or AC. To mitigate selection bias, PSM was performed on both groups. Our analysis revealed no statistically significant difference in OS between patients who underwent NAC and those who received AC, both before and after PSM. Notably, after PSM, the original differences in CSS between the two groups were also eliminated. These findings indicate that NAC does not seem to confer long-term survival advantages than AC in patients with HR+ MBC. Consequently, caution and careful consideration should be exercised when selecting patients for NAC in the HR+ MBC context. The therapeutic efficacy of antitumor drugs is contingent upon a multitude of mechanisms (41). The potential reasons why NAC may not confer long-term benefits to HR+ MBC in this study include: the majority of the enrolled population consisted of patients with T1/2 (93%), N0 (91.6%), and AJCC stage I or IIa, indicating a relatively early stage of the disease. Moreover, all patients had HR+ MBC, which typically has a favorable prognosis, with 5-year overall OS and BCSS rates exceeding 90%. Under these circumstances, the advantages of NAC may not be readily apparent. In this cohort, we identified 707 patients who underwent NAC, a sample size that is relatively small and may not fully reflect the value of NAC in the overall MBC population. Regarding this issue, we plan to collect more patients with MBC who have received NAC in the future and conduct further stratified analyses. Through subgroup analyses, we aim to identify specific populations that may benefit from NAC.

In this study, we successfully developed and validated a prognostic risk prediction model for HR+ MBC, which exhibited reliable predictive ability. Nonetheless, several limitations should be acknowledged. Firstly, this retrospective study relied on the SEER database, and therefore, there may be inherent selection bias in data screening. Further validation through additional clinical data and prospective studies is warranted. Secondly, endocrine therapy represents a vital treatment modality for HR+ MBC; however, the SEER database lacked information regarding its administration, thus precluding its integration into the analysis. Additionally, the number of my external validation queues is relatively small, we plan to collaborate with other hospitals in subsequent studies to conduct multicenter data collection and enhance the validation power. Finally, for patients receiving NAC and AC, it would be valuable to conduct further stratified analyses considering tumor size and lymph node metastasis, in order to more accurately identify the population that would benefit from chemotherapy.

## 5 Conclusion

In summary, this study successfully constructed and verified a nomogram model to predict the survival of HR+ MBC patients. Age, race, marital status, mass size, metastasis, and treatment modality were identified as independent prognostic factors. Our study offers valuable insights into prognosis prediction and clinical decision-making for HR+ MBC patients. Furthermore, the study demonstrated that NAC does not confer long-term survival benefits than AC in HR+ MBC patients and should be carefully considered on an individual basis prior to surgery.

## Data Availability

The raw data supporting the conclusions of this article will be made available by the authors, without undue reservation.
